# A Factor in Disease Control

**Published:** 1920-04-10

**Authors:** 


### A FACTOR IN DISEASE CONTROL.

In the continued discussion of how best to prevent, detect, and limit the spread of venereal disease, there is one point upon which relatively little emphasis has been laid. This is the great potential value of sympathetic co-operation in this matter by members of the dental profession. The opportunities which inevitably occur to them and the frequency with which mouth, pharyngeal or other manifestations of the disease may come under their notice is a matter which need not, in a professional journal, be fully discussed. But appreciation of the fact that the dentist can play an all-important part in the work was exemplified by the surgeon-general of the United States Public Health Service in sending, during the earlier stages of the anti-venereal campaign, a circular letter, a bulletin, and a pledge card to every dentist in the country. Therein was clearly described the especial importance of interesting the dental profession, since syphilis might undoubtedly be transferred to innocent persons through the medium of dental operations, and outline was also made of a variety of other dental responsibilities. The letter concluded "An agreement card is enclosed. Please sign and mail to-day; no stamp is required." Within a relatively short time the authorities were able to report that a high percentage had officially joined the movement, and in doing so pledged themselves, not only to lend their full co-operation to the scheme, but also to report all venereal cases which came under their notice, and to advise the sufferers to seek proper treatment for their condition. We understand that considerable good resulted and that numbers of cases were thus brought more rapidly under control. A similar agreement procedure was adopted in the case of campaigns carried out among physicians, druggists, and advertising media, and gives a graphic instance of the direct action principle, to which, in the case of our own anti-venereal crusade, not a few authorities in this country would do well to give more consideration.

It is true that patients having lesions in the mouth, who know or suspect they have syphilis and are likely to admit it, do not as a rule seek the services of a dentist, unless compelled to do so. Our public educational methods have probably achieved this much at least; but if one may judge from the average hospital clinic, many who have the lesions but are relatively unaware of them, present themselves unhesitatingly for extractions, etc.

Therefore we have at least a further method of finding unsuspected syphilitic cases, but there is another aspect of the problem which, although our words do not apply to the number of able, fully-trained dental surgeons which this country fortunately possesses, creates an urgent need for improved clinical knowledge among many of those who practise dentistry. Syphilis is no respecter of persons; neither is it a respecter of tissues. Simple scaling of the teeth may, and usually does, involve a breaking in the continuity of the gum tissue, with production of a focus possible of infection by an unclean instrument. Neither the possibly infective nature of saliva nor the absolute necessity for study and strict observance of every principle of asepsis can be exaggerated. Without this knowledge the unsuspecting syphilitic becomes an obvious menace to the health of a community. And let us remember that syphilis is not by any means the only disease transferrable from mouth to mouth. We could give numberless examples of timely and valuable advice given to patients by their dentist. Always to recognise the various lesions significant of disease, which may be found in the mucous linings of the mouth, requires considerable knowledge and experience, and all credit to those members of the dental profession who have efficiently acquainted themselves with their meaning.

As the American pamphlet says: on every hand dentists have been confronted with increased responsibility in the matter of general health, not merely because they have aspired to greater things, but because it has been thrust upon them, and the proposition must be met face to face.

